# Illness Beliefs Predict Mortality in Patients with Diabetic Foot Ulcers

**DOI:** 10.1371/journal.pone.0153315

**Published:** 2016-04-20

**Authors:** Kavita Vedhara, Karen Dawe, Jeremy N. V. Miles, Mark A. Wetherell, Nicky Cullum, Colin Dayan, Nicola Drake, Patricia Price, John Tarlton, John Weinman, Andrew Day, Rona Campbell, Jenna Reps, Daniele Soria

**Affiliations:** 1 Division of Primary Care, University of Nottingham, Nottingham, NG7 2RD, United Kingdom; 2 School of Social and Community Medicine, University of Bristol, Bristol, BS8 2PS, United Kingdom; 3 Rand Corporation, Santa Monica, CA, 90401–3208, United States of America; 4 Department of Psychology, University of Northumbria, Newcastle, NE1 8ST, United Kingdom; 5 School of Nursing, Midwifery & Social Work, University of Manchester, Manchester, M13 9PL, United Kingdom; 6 Institute of Molecular & Experimental Medicine, University of Cardiff, Cardiff, CF14 4XN, United Kingdom; 7 Department of Podiatry, Southmead Hospital, Bristol, BS10 5NB, United Kingdom; 8 School of Healthcare Studies, Cardiff University, Cardiff, CF14 4XN, United Kingdom; 9 Matrix Biology Research Group, University of Bristol, Bristol, BS40 5DU, United Kingdom; 10 Institute of Pharmaceutical Sciences, King's College London, London, SE1 9RT, United Kingdom; 11 Department of Clinical Biochemistry, University Hospitals Bristol, Bristol, BS1 3NU United Kingdom; 12 School of Computer Science & Advanced Data Analysis Centre, University of Nottingham, Nottingham, NG8 1BB, United Kingdom; Kings College, UNITED KINGDOM

## Abstract

**Background:**

Patients’ illness beliefs have been associated with glycaemic control in diabetes and survival in other conditions.

**Objective:**

We examined whether illness beliefs independently predicted survival in patients with diabetes and foot ulceration.

**Methods:**

Patients (n = 169) were recruited between 2002 and 2007. Data on illness beliefs were collected at baseline. Data on survival were extracted on 1^st^ November 2011. Number of days survived reflected the number of days from date of recruitment to 1^st^ November 2011.

**Results:**

Cox regressions examined the predictors of time to death and identified ischemia and identity beliefs (beliefs regarding symptoms associated with foot ulceration) as significant predictors of time to death.

**Conclusions:**

Our data indicate that illness beliefs have a significant independent effect on survival in patients with diabetes and foot ulceration. These findings suggest that illness beliefs could improve our understanding of mortality risk in this patient group and could also be the basis for future therapeutic interventions to improve survival.

## Introduction

The psychological functioning of patients with diabetes has been shown to be of clinical importance. For example, indices of psychological functioning have been associated with poorer metabolic control[1]; greater treatment non-adherence[2] and an increased risk of diabetic complications.[3] Research with patients with diabetic foot ulcers (a lesion in the skin which penetrates the dermis and occurs below the ankle) has also been suggestive of a role for psychological factors in predicting clinical outcomes. For example, in patients with, or at risk, from foot ulceration, depression has been associated with an increased risk of ulceration,[4] delays in the rate of ulcer healing[5] and a 2 fold greater risk of mortality.[6]

The evidence regarding the relationship between psychological functioning and outcomes in patients with foot ulcers is, however, limited in two main ways. First, it has largely focussed on depression and second, the evidence pertaining to the role of depression is equivocal. For example, contrary to the studies cited above, data exist which suggest that depression is not related to ulcer recurrence[4,6] or amputation.[7] Similarly, the effect of depression on ulcer healing has been shown not to withstand adjustment for clinical predictors.[8] These observations lead us to speculate that a focus on depression alone may be limiting our understanding of the ways in which psychological functioning can influence clinical outcomes in diabetic foot ulceration; and that it may be necessary to examine the role of other psychological processes.[5,9]

If we are to extend our assessment of psychological factors beyond depression, which factors are worthy of further enquiry? The influential self-regulatory model of illness[10] can inform this question. The model asserts that patients form illness beliefs when contending with a health threat and that these beliefs play a central role in determining patients’ emotional and behavioural responses to their illness. According to the current taxonomy patients’ beliefs centre around the following core constructs: identity (beliefs regarding the experience of symptoms associated with the illness); consequences (beliefs regarding the outcomes of the illness); timeline (beliefs regarding the likely duration of the illness); personal control (beliefs regarding one’s ability to influence the course of the illness); treatment control (beliefs regarding the effectiveness of treatment to cure or control the illness); coherence (beliefs regarding one’s understanding of the illness); emotional representations (beliefs regarding the emotional impact of the illness) and causal representations (beliefs regarding the cause of the illness). These beliefs are not held in isolation, but are hypothesised to interact with each other to form an overall illness schema. The relationship between beliefs and outcomes is necessarily influenced by the nature of the disease in question; and as such, specific beliefs or belief schema, are not universally associated with positive or adverse outcomes.

Thus, while emotional responses, such as depression, arise in direct response to illness and the threat of illness, illness beliefs are also influential in determining these emotional responses and could, therefore, be expected to influence outcomes in patients with diabetic foot ulcers. Evidence in support of this comes from the wider literature on patients with diabetes. For example, a recent systematic review provides evidence in support of illness beliefs being associated with glycaemic control in diabetes.[11] Furthermore, a study comparing the effects of depression versus illness beliefs in predicting dietary, quality of life and glycaemic control outcomes in diabetes, showed that illness beliefs were more consistent and stronger determinants of these outcomes than depression.[12] Evidence pertaining specifically to patients with diabetic foot ulcers has shown that illness beliefs are important determinants of self-care, in particular foot-care practices: with patients identity, personal control and coherence beliefs found to predict engagement with foot self-care.[13]

Of further relevance here is recent work with other patient groups which has shown that illness beliefs predict mortality. For example, van Dijk and colleagues[14] reported in a cohort of patients with end stage renal disease that beliefs regarding treatment control predicted mortality: with death being more likely in patients who believed their treatment to be less effective. Similar findings were reported by Chilcot, Wellstead and Farrington (2011) who also found negative beliefs about the effectiveness of treatment predicted mortality in patients with end stage renal disease.[15] More recently, Crawshaw, Rimington, Weinman and Chilcot (2015) reported that changes in illness perceptions, specifically a change from positive to negative beliefs, predicted mortality in patients who had undergone cardiac valve replacement.[16]

In sum, the evidence regarding the role of depression in predicting clinical outcomes in patients with diabetic foot ulcers has been equivocal. In contrast, the emerging evidence on illness beliefs from patients with diabetes and other chronic conditions suggests they may be influential in predicting a range of clinical outcomes, including mortality. We sought to add to this literature by considering the role of depression and illness beliefs in predicting mortality in patients with type 1 and type 2 diabetes and with a diabetic foot ulcer. In line with previous research we hypothesised that the time to death would be shorter in patients with negative beliefs[16]; and we specifically expected to find that beliefs regarding symptoms, personal control and/or coherence would be related to mortality in view of their role in predicting foot self-care in this patient group.[13] Although, in view of the, as yet, limited evidence in this area, we did not hypothesise as to the direction of these effects. Furthermore, in view of the prominence of depression as a predictor of mortality in diabetes [6,7,17] our predictive models were constructed to examine whether illness beliefs predicted mortality after examining the role of potential demographic and clinical determinants and depression.

## Research Design and Methods

### Procedure

Patients participated in a prospective observational study. At baseline, the following clinical and demographic data were collected on all participants: age, gender, glycated haemoglobin (HbA1c), number of previous ulcers, presence/absence of infection in ulcer, diabetes type, neuropathy and ischemia and ulcer size. Participants also completed self-report measures of illness beliefs,[18] and depression[19] at baseline. Data on survival were collected after the survival census point (1^st^ November 2011).

### Patients

A convenience sample of patients with type 1 or type 2 diabetes mellitus and a foot ulcer was recruited from outpatient podiatry clinics in secondary care in the UK between January 2002 and January 2007. Patients were recruited into a longitudinal research programme examining psychological and behavioural aspects of diabetic foot ulceration. This study was approved by the North Somerset & South Bristol Research Ethics Committee and all participating patients provided written informed consent.

All clinics subscribed to a standard regimen of foot care, i.e., aggressive debridement at each visit, treatment of infections with antibiotics and the use of removable Scotch-casts and other footwear/devices for offloading ulcers on weight-bearing areas, minimising the likelihood of between-centre variations in treatment outcomes. Inclusion/exclusion criteria ensured the population consisted of patients with neuropathic or neuroischaemic ulcers. Patients were not eligible if they had: no palpable pulses on the affected foot; a history of major amputation (i.e., any lower limb amputation greater than a single digit); known large vessel peripheral vascular disease (e.g., previous bypass surgery, angioplasty); advanced diabetic retinopathy with severe visual impairment; advanced nephropathy (e.g., on dialysis); other severe disabling medical conditions (e.g., stroke); or were being treated with platelet-derived growth factor, tissue engineered skin or total contact casts.

One hundred and sixty-nine patients were recruited. In November 2011, survival data (i.e., deceased versus alive at 1/11/11; and, if deceased, date of death) were requested from General Practitioners. Data were available for 160 patients.

### Measures

#### Illness beliefs

Participants completed the Brief Illness Perceptions Questionnaire (BIPQ)[18] derived from the self-regulatory model of illness.[10] This instrument is recommended in studies involving older participants and/or ill participants and so was selected for the present study. The instrument captured patients’ beliefs regarding their foot ulcer in the following domains: identity (‘How much do you experience symptoms?’); consequences (‘How much does your ulcer affect your life?’); timeline (How long do you think your ulcer will continue?); personal control (‘How much control do you feel you have over your ulcer?’); treatment control (‘How much do you think your treatment can help your ulcer?’); coherence (‘How well do you feel you understand your ulcer?’) and emotional response (‘How much does your ulcer affect you emotionally?’). The cause and concern items from this scale were excluded. The causal item involves a response format that differs from the other belief items. Specifically, patients respond to an open ended question with free text to indicate up to 3 causes of their condition. As such no score is obtained and thus it cannot be analysed in the same way. It is, therefore, common for researchers not to consider causal beliefs in quantitative analyses. Indeed, this is the case for all of the previous studies which have looked at the role of illness beliefs in predicting mortality.[14–16] With regard to the concern item, this is one of two items in the BIPQ which capture emotional representations. In view of the age and frailty of our participants, we included just one of the items exploring emotional representations as this allowed us to examine this aspect of the model whilst limiting participant burden. The range of scores for each subscale was 0–10, with higher scores indicating a stronger belief in the relevant domain. The reliability, concurrent and predictive validity of the instrument has been reported elsewhere.[18,20]

#### Depression

Depression was measured using the depression subscale of the Hospital Anxiety and Depression Scale (HADS).[19] The range of scores for this subscale was 0–21, with higher scores reflecting higher levels of depression. The Cronbach’s alpha reliability coefficient for the subscale in the present study was 0.849.

#### Glucose control

HbA1c was measured at baseline. This provides a surrogate marker of disease control by providing an average of blood glucose levels in the previous 8–12 weeks. HbA1c was measured by cation exchange high performance liquid chromatography using a Menarini HA-8140 analyser and associated reagents (A. Menarini Diagnostics, Wokingham, UK). The assay was maintained in alignment with the Diabetes Control and Complications Trial method,[21] with no significant assay drift and a between-batch imprecision (CV) of 1.8% (at mean HbA1c 5.5% [37 mmol/mol]). All assays were performed on the same instrument.

#### Neuropathy and ischaemia assessments

Neuropathy was assessed by applying a 10g nylon monofilament to a number of sites on the affected foot and patients reporting the presence/absence of sensation. Level of neuropathy was based upon the number of tested sites with sensory loss. Percentage rather than absolute values were used as the number of sites assessed varied between podiatrists. Ischaemia was assessed by measuring number of palpable pulses at the dorsalis pedis and posterior tibial areas of the affected foot. All assessments were conducted by the treating podiatrist at each centre.

#### Ulcer assessments

Data were collected from clinical records on all patients regarding the number of previous ulcers, the size of the presenting ulcer and the presence/absence of infection in the presenting ulcer. The assessment of ulcer size involved placing a disposable transparent film over the ulcer and tracing the topical area of the ulcer. The tracing was then placed on a digital tablet (Visitrack: Smith and Nephew, London, UK) and the area of the ulcer was re-traced with a stylus to produce a measurement of absolute ulcer area (in mm^2^). These assessments were conducted by the treating podiatrist at each centre.

### Statistical methods

Pearson’s correlations were computed to examine the inter-relationships between the individual illness belief domains. One way analysis of variance and chi-square analysis were conducted to compare patients with and without missing survival data on all predictor variables. After checking that assumptions were satisfied, survival analysis was undertaken using Cox regression models to examine the predictors of time to death. The survival outcome was number of days survived from the date of recruitment to the census point (1/11/11) or death from any cause. The survival analysis involved two stages. In the first, all potential clinical and demographic predictors and depression were examined in univariate analyses to identify significant predictors. In the second step, all seven belief measures were added to only those covariates identified as significant in the first step. This is in keeping with the self-regulatory model[10] which argues that a patient’s understanding of their illness, and subsequent behavioural and emotional responses, are influenced by their belief schema, i.e., all of the belief domains represented in the model. Although this resulted in our models having up to ten predictors, this approach is in keeping with contexts in which it is appropriate to relax the rule of ten predictors per number of outcomes[22];

As both the predictor and outcome variables contained missing values, imputation methods were used to maximise the available data for the survival analysis. The independent variables appeared to be missing completely at random: Little’s test[23] returned a p-value of 0.74. As only 79 out of the 160 patients contained no missing values, we imputed the missing predictor values using k-nearest neighbours, with k = 5, to ensure there was sufficient power.[24] For the outcome measures, survival status was known for 160 patients. Of these, 24 were known to have died, but their date of death was unknown. Thus, we performed multiple imputations to estimate the survival time for these patients. Simulation studies[25,26] have shown that the required number of repeated imputation methods can be as low as three for data with 20% of missing entries. In the present work we took a conservative approach and used five imputation techniques with 15% of missing data. The first imputation method considered the patients to survive midway between their inclusion into the study and study end date. The second identified the average proportion of time between patients’ start dates and the study end date for all the patients who died with a known date of death and estimated the patient’s death to be the same ratio between their start date and the study end date. The third imputed survival time was based on the survival time of the patient with the closest start date and the fourth survival time was based on the survival time of the four patients with the closest start date. The fifth survival time was based on the average of the previous four survival times. The Cox survival analysis was performed using all five predicted survivals. Our primary analyses, therefore, focused on the imputed dataset using the fifth survival time, as this captured all previous imputations. However, we report results from the non-imputed dataset and the datasets using the 4 other imputation methods in order to examine the robustness of our findings.

Missing imputation methods were implemented using R, a free software environment for computing and graphics.[27] All other analyses used SPSS, Version 21.

## Results

### Cohort Characteristics

Of the 160 patients for whom data on mortality were available, n = 104 were alive at the census point (cumulative survival rate at year 1 was 0.926); n = 32 deceased and date of death known; and n = 24 deceased and date of death not known. No data were available from general practitioners for the remaining 9 patients (no additional information given) and these patients were excluded from the survival analyses. Analyses were conducted to compare patients with and without survival data on all the predictor variables. No differences were evident between the groups on any variable (data not shown), with the exception of age which approached significance (p = 0.056): patients with missing survival data were older (mean = 65 years) compared with patients with complete data (mean = 60 years).

Table 1 shows that the median survival period was 6.4 years; the average age of participants was 61 years; most patients had a diagnosis of type 2 diabetes (n = 111) and, in keeping with the known prevalence of these ulcers, two-thirds of our participants were male. The clinical data indicated moderately high levels of neuropathy and ischemia and average HbA1c levels suggested poor glucose control. Most patients had had an ulcer previously and for approximately one-third of patients the index ulcer was infected at study entry. The psychological data revealed, on average, low levels of depression. The illness beliefs measure indicated that patients reported that they experienced few physical symptoms associated with their ulcers (identity beliefs); believed their ulcers had significant consequences for them (consequence beliefs); and were likely to last a moderately long time (timeline beliefs). Patients also reported moderate levels of personal control over their ulcers (personal control beliefs), but had a greater belief in the effectiveness of treatment (treatment control beliefs). Coherence beliefs suggested that patients’ perceived they had a moderately good understanding of their ulcers and also believed that their ulcers affected their emotional well-being. Pearson’s correlations between the individual belief subscales (non-imputed data) revealed a reasonable degree of inter-correlation between the subscales, with all subscales correlating with at least one other subscale. The only exception to this was the measure of illness coherence (see Table 2).

**Table 1:** Clinical, demographic and psychological characteristics of the cohort.

	*Mean (standard deviation) / Frequency	Available data (N)
Median survival (days)	2351 (+/-912)	136
Gender	120 male / 40 female	160
Age	61.21 (+/-11.85)	160
HbA1c % [mmol/mol]	8.69 (+/-1.85); [71.5]	150
Number of previous ulcers	1 (+/-2)	138
Ulcer infected at baseline	61 yes / 98 no	159
Diabetes type 1/2	Type 1 = 45/Type 2 = 111	156
Ulcer area at baseline (mm^2^)	17.35 (32.63)	159
Neuropathy score (%)	74 (+/-32)	152
Ischemia score (%)	70 (+/-35)	154
Depression	5.60 (+/-4.17)	128
Identity beliefs	2.90 (+/-2.82)	119
Consequence beliefs	6.42 (+/-2.16)	115
Timeline beliefs	5.98 (+/-1.84)	119
Personal control beliefs	6.16 (+/-2.42)	116
Treatment control beliefs	8.20 (+/-1.33)	117
Coherence beliefs	6.10 (+/-2.19)	119
Emotional response beliefs	5.45 (+/-2.71)	119

* Mean scores reported unless specified otherwise

**Table 2:** Pearson’s product moment correlations between illness belief subscales.

	Consequence beliefs	Timeline beliefs	Personal control beliefs	Treatment control beliefs	Identity beliefs	Coherence beliefs
Timeline beliefs	0.350 p<0.0001					
Personal control beliefs	-0.234 p = 0.014	-0.155 p = 0.099				
Treatment control beliefs	-0.091 p = 0.337	-0.029 p = 0.755	-0.003 p = 0.978			
Identity beliefs	0.539 p<0.0001	0.394 p<0.0001	-0.305 p = 0.001	0.092 p = 0.327		
Coherence beliefs	0.016 p = 0.863	0.079 p = 0.392	-0.030 p = 0.751	0.078 p = 0.406	0.071 p = 0.446	
Emotional response beliefs	0.510 p<0.0001	0.274 p = 0.003	-0.140 p = 0.134	0.211 p = 0.023	0.302 p = 0.001	-0.030 p = 0.744

### Examining predictors of time to death

The first step in the univariate Cox regression models involved examining the role of potential clinical and demographic predictors and depression. The results revealed that only diabetes type (1/2) and ischemia were significant predictors of time to death (see Table 3). In the multivariate model, the measures of illness beliefs were added to these significant covariates. These results showed that ischemia remained a significant predictor of time to death (HR 0.976, p<0.0001) and that coherence (HR 0.775, p = 0.036) and identity beliefs (HR 1.245, p = 0.036) also emerged as significant predictors, with treatment control beliefs (HR 0.735), p = 0.086) falling below the threshold of significance. Specifically, patients with less ischemia; a poorer understanding of their condition; who perceived they experienced more symptoms; but also a greater belief in the effectiveness of treatment were most likely to die (see Table 3).

**Table 3:** Cox regression analyses examining predictors of time to death.

Univariate analyses				^1^Multivariate analysis			
Covariate	Hazard ratios	p	95%CI	Covariate	Hazard ratios	p	95%CI
Age	1.021	.179	.990–1.053	Diabetes 1/2	.395	.107	.128–1.223
Gender	1.029	.945	.462–2.291	Ischemia score	.976	.000	.965-.987
Ulcer area at baseline (mm^2^)	1.003	.585	.993–1.012	Consequence beliefs	.959	.817	.671–1.370
Ulcer infected at baseline	.792	.512	.394–1.592	Timeline beliefs	.993	.965	.717–1.374
Diabetes 1/2	.304	.026	.107-.868	Personal control beliefs	1.085	.465	.872–1.351
Number of previous ulcers	1.086	.182	.962–1.227	Treatment control beliefs	.735	.086	.517–1.045
HbA1c	.869	.181	.708–1.067	Identity beliefs	1.245	.036	1.014–1.529
Depression	.975	.579	.892–1.066	Coherence beliefs	.775	.036	.610-.983
Neuropathy	1.005	.381	.994–1.017	Emotional response beliefs	.890	.274	.722–1.097
Ischemia score	.975	< .0001	.966-.985				

^1^ Multivariate analysis included only significant covariates identified in univariate analyses and all illness beliefs

These analyses were repeated following imputation of missing predictor and outcome data as described above, and the results remained largely unchanged. In particular, regardless of which of the 5 imputation methods were used on the time to death variable, the univariate analyses revealed that only the measures of ischemia, diabetes type (1/2) and age were significant independent predictors of time to death (data not shown). Similarly, the multivariate analyses which included the illness belief measures revealed that for all 5 imputation methods, only ischemia and identity beliefs were significant predictors (see Table 4).

**Table 4:** Cox regression analyses using imputed data to examine effects of significant clinical and demographic covariates and illness beliefs on time to death.

	Imput-ation 1			Imput-ation 2			Imput-ation 3			Imput-ation 4			Imput-ation 5		
Covariate	HR	p	95%CI	HR	p	95%CI	HR	p	95%CI	HR	p	95%CI	HR	p	95%CI
Age	1.283	.094	.958–1.717	1.315	.065	.983–1.760	1.257	.126	.938–1.684	1.252	.130	.936–1.676	1.272	.108	.949–1.705
Diabetes 1/2	0.602	192	.281–1.291	0.591	.175	.277–1.263	0.613	.211	.2858–1.320	0.583	.173	.269–1.266	0.607	.203	.282–1.309
Ischemia	.425	.000	.313-.578	.420	.000	.308-.573	.418	.000	.308-.566	.405	.000	.298-.551	.415	.000	.305-.564
Consequence beliefs	.732	.176	.465–1.151	.706	.133	.448–1.112	.848	.466	.545–1.32	.828	.407	.530–1.293	.807	.342	.518–1.257
Timeline beliefs	1.280	.236	.851–1.925	1.250	.279	.834–1.873	1.189	.381	.808–1.75	1.157	.460	.786–1.702	1.24	.286	.835–1.841
Personal control beliefs	1.277	.261	.834–1.954	1.276	.264	.833–1.954	1.206	.367	.803–1.812	1.182	.415	.791–1.766	1.228	.333	.810–1.860
Treatment control beliefs	.770	.145	.542–1.094	.746	.101	.526–1.059	.790	.186	.557–1.121	.799	.209	.563–1.134	.783	.173	.550–1.113
Identity beliefs	1.809	.017	1.113–2.940	1.995	.007	1.213–3.281	1.622	.050	1.001–2.628	1.654	.040	1.022–2.675	1.669	.038	1.028–2.71
Coherence beliefs	.796	.227	.550–1.152	.769	.161	.532–1.111	.828	.317	.573–1.198	.796	.220	.552–1.146	.808	.260	.557–1.172
Emotional response beliefs	.729	.120	.489–1.086	.733	.129	.491–1.095	.756	.156	.514–1.113	.737	.128	.497–1.092	.741	.134	.500–1.097

## Conclusions

We examined the role of illness beliefs in predicting time to death in patients with diabetic foot ulcers; controlling for other potential clinical and demographic determinants. These analyses were conducted with and without imputation of missing data. The results from the multivariate models, without imputation, revealed that ischemia, coherence and identity beliefs predicted time to death. Specifically, death occurred more quickly in individuals with less ischemia, who perceived their ulcers were associated with greater symptoms and had a poorer understanding of their condition. When these analyses were repeated with imputation of missing data for both predictor and outcome variables, the findings were largely unchanged, with degree of ischemia and identity beliefs emerging as significant predictors of both mortality and time to death in all analyses. In view of the increased power associated with the imputed datasets, the discussion of our findings will focus, primarily, on these results.

Our findings have several implications. First, they add to an existing literature which has shown that patients’ illness beliefs can influence clinical outcomes in diabetes (e.g., quality of life, glycaemic control[11,12]): with the present work identifying an independent role for illness beliefs in predicting survival. These results are also in keeping with findings from other patient groups[15,16] and a recent systematic review[28] all of which have shown how beliefs regarding one’s illness are predictive of mortality over periods as short as 1.32 years[15] and as long as 10 years.[16]

Second, these results suggest that approaches to understanding mortality risk in this patient group[29] may be improved through the inclusion of illness beliefs in risk models. Our data showed that, even after controlling for other predictors, illness beliefs predicted survival; and that identity beliefs emerged as being of particular importance. Indeed, evidence suggesting that illness beliefs are not only modifiable, but that illness belief based interventions can produce significant changes in a range of outcomes (e.g., adherence behaviours, mood, return to work) and across many different diseases, including diabetes [30–33]; suggests that the measurement of illness beliefs may not only improve our understanding of the risk factors associated with mortality, but could also be incorporated into interventions to improve survival. Although detailed consideration of the features and mechanisms of such an intervention is beyond the scope of this paper, it could be hypothesised that evidence identifying significant relationships between illness beliefs and glycaemic control[11] and illness beliefs and self-care behaviours[13] suggests that any such intervention could improve survival via these pathways.

The third issue concerns the seemingly central role of identity beliefs in predicting mortality. Identity beliefs are concerned with an individual’s perception of the extent to which their condition is symptomatic and are often associated with more favourable outcomes (e.g., better adherence, attendance at cardiac rehabilitation, etc.[34,35] In contrast, in the present study the experience of greater foot ulcer symptoms was associated with a faster, not slower, time to death. This finding could simply reflect the fact patients with greater symptoms had more severe disease which resulted in the greater risk of mortality. This explanation, however, is not consistent with what is known about neuropathic and neuroischemic foot ulcers. Nerve damage is a defining feature of such ulcers. Consequently, the more severe the underlying pathology, the more likely the patient will experience fewer symptoms. Thus, if we were considering a pathophysiological explanation alone, then the experience of fewer symptoms (an indication of more severe disease) might be associated with a faster time to death, rather than the converse.

Our data clearly do not allow us to delineate precisely why patients who believe their ulcers are associated with greater symptoms might experience a faster time to death. But it may be possible to speculate to possible pathways based on the relationship between identity beliefs and the other belief domains. In particular, significant positive associations were evident between identity beliefs and consequence, timeline and emotional response beliefs; as well as a significant negative correlation with personal control beliefs. Thus, patients who perceived their ulcers were associated with greater symptoms also believed that their ulcers had more serious consequences for them, would last a long time, that they were associated with greater emotional distress and less personal control. This constellation of beliefs may have led to unhelpful behavioural and/or emotional responses (e.g., poorer adherence to treatment) leading to the observed association with mortality.

We acknowledge that our understanding of the relationship between identity beliefs and mortality is significantly compromised by the fact that our understanding of patients’ beliefs was limited to responses to a single item which does not give us insight into the types of symptoms that patients were considering when responding. Future work, using either qualitative methods,[36] and/or which capture illness beliefs using more detailed methods such as the Illness Perceptions Questionnaire—Revised[37] could help to illuminate this relationship between identity beliefs and mortality.

A further related issue concerns the fact that previous work looking at the relationship between illness beliefs and mortality has identified a role for treatment control beliefs, not identity beliefs, in predicting mortality.[14,15] This may be due to differences in the disease characteristics of the patient groups: both of the cited studies focused on patients with renal disease. However, it is also possible the divergent findings relate to differences in the statistical approaches taken. Both studies examined the effects of each illness belief dimension individually in univariate analysis and then, added the significant belief(s) (which in both studies was treatment control) to an adjusted model which included the significant clinical and demographic covariates. This alternative approach, although wholly appropriate, does not permit consideration of the effects of the entire belief schema; and it is possible that the effects of identity beliefs are only evident when the schema is considered.

A fourth issue relates to our finding that ischemia was associated with a survival advantage. As with identity beliefs, ischemia was found to predict time to death in all analyses, thereby suggesting that, although counterintuitive, it was a robust finding. However, a number of issues should be considered when interpreting this result. First, our approach to measuring ischemia involved a single assessment (i.e., the measurement of the number of palpable pulses). However, the accurate measurement of ischemia requires multiple, not single, methods.[38] Thus, our approach, while pragmatic (we selected one method which could be conducted rapidly across all clinics), lacked precision and this may have contributed to our finding. Second, as stated, our inclusion criteria were intended to enable us to recruit patients with neuropathic or neuroischaemic ulcers i.e., patients with no palpable pulses (severe ischemia) were excluded. As a result, the patients in this cohort with the greatest levels of ischemia, were likely to be individuals with only moderate ischemic disease; and patients with low levels of ischemia likely to be patients experiencing greater neuropathy. As the treatments for microvascular complications such as neuropathy are considered not to be as effective as treatments for macrovascular complications,[39] this might explain the apparent survival advantage in our patients with moderate ischemia. In other words, moderate ischemia in this study may have been a marker of less severe neuropathy thus contributing to the observed relationship with mortality and time to death.

The final issue relates to the observation that depression did not influence survival. This finding is consistent with research showing that the effects of depression on clinical outcomes in diabetes are equivocal.[29] Indeed, our data support a growing literature suggesting that a focus on depression in isolation may not be helpful when considering how psychological factors, and psychological interventions, influence clinical outcomes in diabetes.[12,40,41] In the case of the present work, we were unable to detect a statistically significant independent effect of depression. However, it is worth noting that post-hoc analyses (data not shown) revealed that depression was significantly positively correlated with identity beliefs, thus suggesting the potential for an indirect effect of depression on mortality outcomes.

In summary, our analyses have shown a significant independent effect of patients’ illness beliefs on survival in patients with diabetic foot ulcers. Potential limitations of this work relate to the observational design, the modest sample size, the exclusion of patients for whom we were unable to obtain survival data from clinical records and the limited number of events (deaths) in our cohort. The latter issue was, of course, a function of our data. We did, however, endeavour to make sure we were able to use as much data as possible through the use of multiple imputation methods. Similarly, with regard to our sample size, it is worth noting that, although modest, it was greater than the mean sample size reported in a systematic review of previous work examining the role of illness beliefs in survival[28]; and our excluded patients did not differ from the rest of the cohort on any of the predictors of survival.

A further consideration is that our analyses focussed on patients with predominantly neuropathic or neuroischaemic ulcers and poor glucose control. Although this is common in patients with diabetic foot disease, it does potentially limit the generalisability of our findings beyond this patient group. Finally, our approach to measuring illness beliefs was pragmatic but lacked precision. Although the brief IPQ is particularly suitable for studies with older and/or frail patients, it relies on single items for the measurement of each belief domain and this necessarily precludes a detailed analysis of patients’ beliefs. Notwithstanding these limitations, these results broaden our understanding of the role of psychological processes in diabetes and add to the growing literature suggesting that individuals’ beliefs about their illness may have prognostic significance.

## References

[1] Lustman PJ, Anderson RJ, Freedland KE, de Groot M, Carney RM, Clouse RE (2000) Depression and poor glycemic control: a meta-analytic review of the literature. Diabetes Care 23: 934–942. 10895843 10.2337/diacare.23.7.934

[2] Gonzalez JS, Peyrot M, McCarl LA, Collins EM, Serpa L, Mimiaga MJ, et al (2008) Depression and Diabetes Treatment Nonadherence: A Meta-Analysis. Diabetes Care 31: 2398–2403. 10.2337/dc08-1341 19033420 PMC2584202

[3] de Groot M, Anderson R, Freedland K, Clouse R, Lustman P (2001) Association of depression and diabetes complications: a meta-analysis. Psychosomatic Medicine 63: 619–630. 11485116 10.1097/00006842-200107000-00015

[4] Gonzalez JS, Vileikyte L, Ulbrecht JS, Rubin RR, Garrow AP, Delgado C, et al (2010) Depression predicts first but not recurrent diabetic foot ulcers. Diabetologia 53: 2241–2248. 10.1007/s00125-010-1821-x 20556354

[5] Vedhara K, Miles JNV, Wetherell MA, Dawe K, Searle A, Tallon D, et al (2010) Coping style and depression influence the healing of diabetic foot ulcers: observational and mechanistic evidence. Diabetologia 53: 1590–1598. 10.1007/s00125-010-1743-7 20411235

[6] Winkley K, Sallis H, Kariyawasam D, Leelarathna LH, Chalder T, Edmonds ME, et al (2012) Five-year follow-up of a cohort of people with their first diabetic foot ulcer: the persistent effect of depression on mortality. Diabetologia 55: 303–310. 10.1007/s00125-011-2359-2 22057196

[7] Winkley K, Stahl D, Chalder T, Edmonds ME, Ismail K (2007) Risk factors associated with adverse outcomes in a population-based prospective cohort study of people with their first diabetic foot ulcer. Journal of Diabetes and its Complications 21: 341–349. 17967704 10.1016/j.jdiacomp.2007.09.004

[8] Takahashi PY, Kiemele LJ, Chandra A, Cha SS, Targonski PV (2009) A Retrospective Cohort Study of Factors that Affect Healing in Long-term Care Residents with Chronic Wounds. Ostomy Wound Management 55: 32–37. 19174587

[9] Vedhara K, Beattie A, Metcalfe C, Roche S, Weinman J, Cullum N, et al (2012) Development and preliminary evaluation of a psychosocial intervention for modifying psychosocial risk factors associated with foot re-ulceration in diabetes. Behaviour Research and Therapy 50: 323–332. 10.1016/j.brat.2012.02.013 22459731

[10] Leventhal H, Nerenz DR, Steele DJ (1984) Illness representations and coping with health threats In: Baum A T S, Singer JE, editor. Handbook of Psychology and Health Volume IV: Social Psychological Aspects of Health. Hillsdale, NJ: Erlbaum pp. p219–252.

[11] McSharry J, Moss-Morris R, Kendrick T (2011) Illness perceptions and glycaemic control in diabetes: a systematic review with meta-analysis. Diabetic Medicine 28: 1300–1310. 10.1111/j.1464-5491.2011.03298.x 21418098

[12] Hampson SE, Glasgow RE, Strycker LA (2000) Beliefs versus feelings: A comparison of personal models and depression for predicting multiple outcomes in diabetes. British Journal of Health Psychology 5: 27–40.

[13] Vedhara K, Dawe K, Wetherell MA, Miles JNV, Cullum N, Dayan C, et al (2014) Illness beliefs predict self-care behaviours in patients with diabetic foot ulcers: A prospective study. Diabetes Research and Clinical Practice 106: 67–72. 10.1016/j.diabres.2014.07.018 25112923

[14] van Dijk S, Scharloo M, Kaptein AA, Thong MSY, Boeschoten EW, Grootendorst DC, et al (2009) Patients’ representations of their end-stage renal disease: relation with mortality. Nephrology Dialysis Transplantation 24: 3183–3185. 10.1093/ndt/gfp184 19383834

[15] Chilcot J, Wellsted D, Farrington K (2011) Illness Perceptions Predict Survival in Haemodialysis Patients. American Journal of Nephrology 33: 358–363. 10.1159/000326752 21430374

[16] Crawshaw J, Rimington H, Weinman J, Chilcot J (2015) Illness Perception Profiles and Their Association with 10-Year Survival Following Cardiac Valve Replacement. Annals of Behavioral Medicine 49: 769–775. 10.1007/s12160-015-9695-2 25697136

[17] Iversen MM, Tell GS, Riise T, Hanestad BR, Østbye T, Graue M, et al (2009) History of Foot Ulcer Increases Mortality Among Individuals With Diabetes: Ten-year follow-up of the Nord-Trøndelag Health Study, Norway. Diabetes Care 32: 2193–2199. 10.2337/dc09-0651 19729524 PMC2782976

[18] Broadbent E, Petrie KJ, Main J, Weinman J (2006) The Brief Illness Perception Questionnaire. Journal of Psychosomatic Research 60: 631–637. 16731240 10.1016/j.jpsychores.2005.10.020

[19] Zigmond A, S., Snaith RP (1983) The hospital anxiety and depression scale. Acta Psychiatrica Scandinavica 67: 361–370. 6880820 10.1111/j.1600-0447.1983.tb09716.x

[20] Broadbent E, Wilkes C, Koschwanez H, Weinman J, Norton S, KJ P (2015) A systematic review and meta-analysis of the Brief Illness Perception Questionnaire. Psychology and Health 30: 1361–1385. 10.1080/08870446.2015.1070851 26181764

[21] The Diabetes Control and Complications Trial Research Group (1993) The Effect of Intensive Treatment of Diabetes on the Development and Progression of Long-Term Complications in Insulin-Dependent Diabetes Mellitus. New England Journal of Medicine 329: 977–986. 8366922 10.1056/NEJM199309303291401

[22] Vittinghoff E, McCulloch CE (2007) Relaxing the Rule of Ten Events per Variable in Logistic and Cox Regression. American Journal of Epidemiology 165: 710–718. 17182981 10.1093/aje/kwk052

[23] Little RJA (1988) A Test of Missing Completely at Random for Multivariate Data with Missing Values. Journal of the American Statistical Association 83: 1198–1202.

[24] Gustavo B, Monard M (2002) A Study of K-Nearest Neighbour as an Imputation Method. HIS 87: 251–260.

[25] Rubin DB (1987) Multiple Imputation for Nonresponse in Surveys. New York: John Wiley & Sons.

[26] van Buuren S, Boshuizen HC, Knook DL (1999) Multiple imputation of missing blood pressure covariates in survival analysis. Statistics in Medicine 18: 681–694. 10204197 10.1002/(sici)1097-0258(19990330)18:6<681::aid-sim71>3.0.co;2-r

[27] Maindonald JH, Braun WJ (2010) Data analysis and graphics using R—an example based approach; Edition r, editor. Cambridge: Cambridge University Press.

[28] Parfeni M, Nistor I, Covic A (2013) A systematic review regarding the association of illness perception and survival among end-stage renal disease patients. Nephrology Dialysis Transplantation 28: 2407–2414. 10.1093/ndt/gft194 23737481

[29] Markowitz SM, Gonzalez JS, Wilkinson JL, Safren SA (2011) A Review of Treating Depression in Diabetes: Emerging Findings. Psychosomatics 52: 1–18. 10.1016/j.psym.2010.11.007 21300190 PMC3043600

[30] Petrie KJ, Perry K, Broadbent E, Weinman J (2012) A text message programme designed to modify patients’ illness and treatment beliefs improves self-reported adherence to asthma preventer medication. British Journal of Health Psychology 17: 74–84. 10.1111/j.2044-8287.2011.02033.x 22107110

[31] Broadbent E, Ellis CJ, Thomas J, Gamble G, Petrie KJ (2009) Further development of an illness perception intervention for myocardial infarction patients: A randomized controlled trial. Journal of Psychosomatic Research 67: 17–23. 10.1016/j.jpsychores.2008.12.001 19539814

[32] Broadbent E, Ellis CJ, Thomas J, Gamble G, Petrie KJ (2009) Can an illness perception intervention reduce illness anxiety in spouses of myocardial infarction patients? A randomized controlled trial. Journal of Psychosomatic Research 67: 11–15. 10.1016/j.jpsychores.2008.11.006 19539813

[33] Jones CJ, Smith HE, Llewellyn CD (2015) A systematic review of the effectiveness of interventions using the Common Sense Self-Regulatory Model to improve adherence behaviours. Journal of Health Psychology. 10.1177/1359105315583372 25957228

[34] Chen S-L, Tsai J-C, Chou K-R (2011) Illness perceptions and adherence to therapeutic regimens among patients with hypertension: A structural modeling approach. International Journal of Nursing Studies 48: 235–245. 10.1016/j.ijnurstu.2010.07.005 20678768

[35] French DP, Cooper A, Weinman J (2006) Illness perceptions predict attendance at cardiac rehabilitation following acute myocardial infarction: A systematic review with meta-analysis. Journal of Psychosomatic Research 61: 757–767. 17141663 10.1016/j.jpsychores.2006.07.029

[36] Beattie A, Campbell M, Vedhara K (2014) “Whatever I do it’s a lost cause.” The emotional and behavioural experiences of individuals who are ulcer free living with the threat of developing further diabetic foot ulcers: a qualitative interview study. Health Expectations 17: 429–439. 10.1111/j.1369-7625.2012.00768.x 22429399 PMC5060729

[37] Moss-Morris R, J W, Petrie KJ, Horne R, Cameron LD, Buick D (2002) The Revised Illness Perception Questionnaire (IPQ-R). Psychology and Health 17: 1–16.

[38] Baker N, Marli-Krishnan S, Fowler D (2005) A user’s guide to foot screening, Part 2: peripheral arterial disease. The Diabetic Foot Journal 8: 58–70.

[39] Fowler MJ (2008) Microvascular and Macrovascular Complications of Diabetes. Clinical Diabetes 26: 77–82.

[40] Ismail K, Winkley K, Rabe-Hesketh S (2004) Systematic review and meta-analysis of randomised controlled trials of psychological interventions to improve glycaemic control in patients with type 2 diabetes. The Lancet 363: 1589–1597. 10.1016/S0140-6736(04)16202-8 15145632

[41] Alam R, Sturt J, Lall R, Winkley K (2009) An updated meta-analysis to assess the effectiveness of psychological interventions delivered by psychological specialists and generalist clinicians on glycaemic control and on psychological status. Patient Education and Counseling 75: 25–36. 10.1016/j.pec.2008.08.026 19084368

